# New strategies of physical activity assessment in cystic fibrosis: a pilot study

**DOI:** 10.1186/s12890-020-01313-5

**Published:** 2020-10-30

**Authors:** Daniela Savi, Luigi Graziano, Barbara Giordani, Stefano Schiavetto, Corrado De Vito, Giuseppe Migliara, Nicholas J. Simmonds, Paolo Palange, J. Stuart Elborn

**Affiliations:** 1 Department of Public Health and Infectious Diseases, “Sapienza” University of Rome, 00185 Rome, Italy; 2 Adult Cystic Fibrosis Centre, Royal Brompton Hospital and Imperial College, London, SW3 6NP UK; 3 Lega Italiana Fibrosi Cistica ONLUS-LIFC, Rome, Italy; 4 Faculty of Medicine, Health and Life Sciences, Queen’s University Belfast, Belfast, UK

**Keywords:** Daily physical activity, Cystic fibrosis, Electronic devices, Accelerometer

## Abstract

**Background:**

Regular physical activity (PA) is a valued part of cystic fibrosis (CF) care. Although the accelerometer, SenseWear Armband (SWA), accurately measures habitual PA in CF, it is mostly used for research purposes. For the first time, we analyzed different methods of measuring PA in daily life by the use of smartphones and other electronic devices such as smartwatch and Fitbit.

**Methods:**

Twenty-four stable adults with CF (mean age 37.5 ± 11.5SD yrs.; FEV_1_ 58 ± 19% predicted, BMI 22.9 ± 3.2) were studied. Daily PA was monitored for seven consecutive days. All patients wore the accelerometer SWA and at the same time they monitored PA with the electronic device they used routinely. They were allocated into one of four arms according to their device: Smartwatch, Fitbit, Android smartphones and iOS smartphones. PA related measurements included: duration of PA, energy expenditure, number of steps.

**Results:**

There was a good agreement between SWA and Fitbit for number of steps (*p* = 0.605) and energy expenditure (*p* = 0.143). iOS smartphones were similar to SWA in monitoring the number of steps (*p* = 0.911). Significant differences were found between SWA and both Smartwatch and Android smartphones.

**Conclusions:**

Fitbit and iOS smartphones seem to be a valuable approach to monitor daily PA. They provide a good performance to measure step number compared to SWA.

**Supplementary information:**

**Supplementary information** accompanies this paper at 10.1186/s12890-020-01313-5.

## Background

Regular physical activity (PA) is a valuable component of cystic fibrosis (CF) care [1, 2] and can enhance quality of life, improve sputum clearance and muscle strength and may also positively influence immune function [3–5]. In addition, there is evidence that PA levels of moderate intensity or greater can increase peak oxygen uptake (V’O2 peak) [6, 7], which is an independent prognostic factor for CF [8]. Specifically, CF patients who spent 30 min per day performing PA above moderate intensity had better exercise tolerance, i.e., higher V’O2 peak [6]. Moreover, supervised exercise intervention studies have demonstrated that regular vigorous exercise can positively impact forced expiratory volume in 1 s (FEV_1_) [9]. Accordingly, exercise training programs have been developed for CF patients that aim to change their lifestyle so as to maintain the positive effects of rehabilitation and increase their ability to undertake daily activities. Recently, it has been also observed that PA improved in CF adults after two years of Lumacaftor/Ivacaftor therapy [10].

PA can be quantified by direct observation and self-reporting questionnaires, and also by assessment of energy expenditure using, for example, motion sensors such as accelerometers [6, 11, 12]. Although studies performed in CF have validated the use of the accelerometer SenseWear Pro3 Armband (SWA) to accurately measure PA [13], it is mostly used for research purposes. Its expense and the need to adequately train staff to interpret and analyze the results are important aspects that reduce its use in clinical practice or self-monitoring daily activities. Recently, it was found that the iPhone was an accurate tool for step counting during various walking conditions in 20 healthy subjects [14]. However, the use of smartphones and other electronic devices such as smartwatches and Fitbit to measure daily physical activity in CF has not been investigated.

The aim of this small-scale, preliminary study was to assess the precision of new electronic devices like Smartwatch, Fitbit, Android smartphone or iOS smartphones in measuring daily physical activity compared to the SWA in an adult CF population. Specifically, we wished to investigate important aspects of electronic devices that are routinely used by CF patients in order to highlight crucial components of a subsequent main study.

## Methods

### Study design

This was an observational, single centre, pilot study and the cohort of 24 patients with CF was recruited at the Policlinico Umberto I Hospital, Sapienza University of Rome, Italy. We assessed whether new electronic devices (i.e. Smartwatch, Fitbit, Android smartphones, iOs smartphones) provide similar information on daily physical activity compared to SWA. Patients were divided into four arms according to the device they routinely used (i.e. Smartwatch, Fitbit, Android smartphones or iOS smartphones).

We used a protocol consisting of: 1) supervised series of 4 physical activity tasks with simultaneous assessment of number of steps and duration of physical activity using their electronic device in an indoor environment over ≤1 h; 2) home monitoring of daily physical activity for seven days (5 weekdays and 2 weekend days) using the SWA and routinely available Smartwatch, Fitbit, Android smartphones or iOS smartphones. All patients wore the SWA and at the same time they monitored daily activity with their Smartwatch, Fitbit, Android smartphones or iOS smartphone. The ethical approval was received from the Policlinico Umberto I Hospital (Sapienza University of Rome, Italy), with approval number 582/11.

### Patients and data collection

Adults attending a CF outpatient clinic were approached for participation in the study from April 2018 to October 2018. They were asked if they usually monitor daily physical activity with an electronic device and what kind of electronic device they usually use among the commercially available Smartwatch, Fitbit, Android smartphones and iOS smartphones. Patients who met the following inclusion criteria were eligible for enrollment: confirmed diagnosis of CF based on either two CF-causing mutations and/or a sweat chloride concentration during two tests of > 60 mmol/l; age ≥ 18 years; FEV1 ≥ 30% predicted; internet access and routine use of either smartphones, androids, smartwatch or a Fitbit to monitor daily physical activity. Patients were excluded from the study if they had at least one of the following exclusion criteria: pulmonary exacerbation within four weeks of the baseline study visit; long-term oxygen therapy; co-morbidities that limited physical activity participation; participation in another clinical trial up to 4 weeks prior to the first baseline visit; pregnancy/ breastfeeding.

After obtaining written informed consent and appropriate screening of medical history, we collected data on age, sex, height, weight, body mass index (BMI), chronic infections and CF comorbidity (pancreas insufficiency, and CF-related diabetes). All patients performed pulmonary function testing at the time of study entry according to American Thoracic Society (ATS) standards and expressed as percentages of predicted values [15].

### Assessment of physical activity

Participants, at the time of the study enrollment, completed a supervised series of 4 physical activity tasks with simultaneous assessment of number of steps and duration of physical activity using their electronic device in an indoor environment over ≤1 h. Static task was supine lying, and active tasks comprised stair-climbing, stationary cycling and walking (modified 6-min walk test (6MWT)). Participants undertook stair-climbing in an indoor stairwell (24 steps), and were instructed to descend and ascend the stairs as they would in everyday life. Participants cycled at heart rate value corresponding to 50% of their predicted maximum heart rate. The 6MWT was performed according to standard protocols [16]. Excluding stair-climbing, all tasks were 6-min in duration.

Daily PA that characterized the lifestyle of the CF patients was assessed at the time of the study enrolment for seven consecutive days (five weekdays and two weekend days). Each patient measured physical activity simultaneously with SWA and with Smartwatch, Fitbit, Android smartphones or iOS smartphones used routinely.

The multi-sensor SWA (BodyMedia, Pittsburgh, USA) has been validated in CF [13, 17]. Studies have shown that the hypersalinity of sweat does not affect the accuracy of energy expenditure estimation, and have demonstrated that the SWA provides an accurate estimate of physical activity in the free-living environment [13, 17]. Patients were instructed to wear the SWA day and night and only to remove it for bathing or showering. The characteristics of SWA were previously reported [6]. The variables measured by SWA were total energy expenditure (Kcal), active energy expenditure (Kcal), PA duration, number of steps and intensity of PA, expressed in metabolic equivalents (METS). Definitions for activity levels based on METS were those used by *Troosters* et al. [7] and are reported on *Supplemental file*. At the same time, each patient measured physical activity with their Smartwatch, Fitbit, Android smartphones or iOS smartphones that was routinely used. Among the Smartwatch group, one patient used Samsung Gear S 3 (Samsung Electronics Co., Ltd., Suwon, South Korea) Tizen OS, 2017 that includes three-axis gyroscope and accelerometer; three patients used Polar M 200 (Electro Oy, Kempele, Finland) Polar FlowSync, 2016 that include GPS and accelerometer; two patients used LG K4 (LG Electronics, Seul, South Korea) 5.1, 2018 that include GPS and accelerometer. Among the Fitbit group, three patients used Samsung Gear Fit 2 (Samsung Electronics Co., Ltd., Suwon, South Korea) Tizen OS, 2017 that includes a three-axis gyroscope and accelerometer; two patients used Fitbit Charge 2 (Fitbit Inc., San Francisco, Ca, USA) proprietary OS, 2017 that contain Global Positioning System (GPS) and triaxial accelerometer; one patient used Huawei Band 2 Pro (Huawei Technologies Co., Shenzen, China) RTOS, 2017 that contains Global Positioning System (GPS) and triaxial accelerometer. Among the Android smartphones group, three patients used Samsung S8 (Samsung Electronics Co., Ltd., Suwon, South Korea) 7.0, 2017 that includes a three-axis gyroscope and accelerometer; two patients used LG P6 (LG Electronics, Seul, South Korea) 7.0, 2017, that include an accelerometer; one patient used Huawei P8 Lite (Huawei Technologies Co., Shenzen, China) 7.0, 2017 that include an accelerometer. Among the iOS smartphones group (Apple Inc., Cupertino, CA, USA) which includes a three-axis gyroscope and accelerometer three patients used iOS 11, 2017; one patient iOS 11.4, 2018; one patient iOS 10, 2016; one patient iOS 12, 2018. The patients were instructed to position correctly the Smartwatch and Fitbit around the wrist all day and night and put Smartphones in their trouser pocket, shoulder bag or backpack. We asked participants to continue their normal daily activities and any respiratory-related medications.

After the study week, all patients returned the SWA and showed the activity data recorded by Smartwatch, Fitbit, Android smartphones or iOS smartphones. To allow comparison between the SWA and Smartwatch, Fitbit, Android smartphone or iOS smartphone data we decided to include in our analysis comparable categories as number of steps, duration of PA and energy expenditure. Physical activity data were recorded for 7 full days for all patients and reported as the average of 7 days for both SWA and Smartwatch, Fitbit, Android smartphones or iOS smartphones.

### Statistical analysis

Descriptive analysis of anthropometric characteristics and clinical outcomes of the 24 CF patients was first carried out to describe the study population at baseline. Differences in continuous outcomes among the four groups were tested with the Kruskal – Wallis test; categorical data are presented as percentages, and comparisons were performed using the Fisher’s Exact test. The Wilcoxon matched-pairs signed-ranks test was performed to analyze differences between accelerometer SWA and each electronic device on energy expenditure (expressed as Kcal), number of steps and duration of physical activity (expressed as min/day). Results were considered statistically significant when the two-sided *P* value was < 0.05. We also performed a concordance analysis of the measurement using the Bland-Altman method. All statistical analyses were performed using the Stata Statistical Software: Release15.1 (StataCorp LLC**,** College Station, TX, USA).

## Results

Twenty-four CF patients (6 in each different device group) were included in this study. Baseline characteristics and pulmonary function data are shown in Table 1. The groups were well matched in terms of age, BMI, lung function and pulmonary infections. Habitual activity levels of the study groups are presented in Table 2.

**Table 1:** Anthropometric characteristics and pulmonary function

Characteristics	All CF (***n*** = 24)	Device 1 (***n*** = 6)	Device 2 (***n*** = 6)	Device 3 (***n*** = 6)	Device 4 (***n*** = 6)
Age, yr	37.6 ± 11.5	33.2 ± 12.1	38.3 ± 8.4	40.7 ± 12.3	38.2 ± 14.3
BMI, Kg/ m^2^	22.9 ± 3.2	24.4 ± 2.5	20.8 ± 4.0	23.6 ± 2.1	22.9 ± 3.6
FEV_1_, % predicted	58.3 ± 19.4	73.2 ± 15.0	52.7 ± 17.1	51.5 ± 11.7	56 ± 26.8
FVC, %predicted	75.7 ± 18.2	88.3 ± 17.0	68.3 ± (15.0)	76.2 ± 14.1	69.8 ± 22.7
*Pseudomonas aeruginosa* colonization, n (%)	16 (66.7)	3 (50.0)	5 (83.3)	5 (83.3)	3 (50.0)
*Staphylococcus aureus* colonization, n (%)	9 (37.5)	3 (50.0)	2 (33.3)	1 (16.7)	3 (50.0)
*Burkholderia cepacia* colonization, n (%)	2 (8.3)	1 (16.7)	0	0	1 (16.7)
Pancreatic insufficiency, n (%)	21 (87.5)	5 (83.3)	5 (83.3)	6 (100.0)	5 (83.3)
CF-related diabetes, n (%)	7 (29.2)	1	1	3	2
F508del Homozygous/Heterozygous	8/15	1/5	4/2	2/4	1/4

Data are presented as mean ± SD, unless otherwise stated. *P <  0.05, differences between CF groups. *Abbreviations*: *BMI* body mass index, *FEV1* Forced expiratory volume in 1 s, *FVC* forced vital capacity, *Device 1* Smartwatch, *Device 2* Fitbit, *Device 3* Smartphone (Android), *Device 4* Smartphone (iOS)

**Table 2:** Daily physical activities measured by the accelerometer SWA

Variable	All CF (***n*** = 24)	Device 1 (***n*** = 6)	Device 2 (***n*** = 6)	Device 3 (***n*** = 6)	Device 4 (***n*** = 6)
Total energy expenditure, kcal	2718.1 ± 955.0	2913.6 ± 940.3	2468.1 ± 681.2	3135.3 ± 1155.0	2314.5 ± 742.0
Active energy expenditure, kcal	1161.7 ± 682.8	1197.8 ± 719.0	1302.2 ± 458.4	1428.1 ± 849.5	692.7 ± 323.0
Steps, number/day	7793.3 ± 5584.8	9478.5 ± 5251.1	7008.7 ± 3945.1	9941.7 ± 7056.2	4512.9 ± 3683.9
Duration Physical Activity, min/day	278 ± 161	237 ± 129	382 ± 157	328 ± 171	159 ± 70
Average METs	2.0 ± 0.4	1.9 ± 0.4	2.3 ± 0.4	2.1 ± 0.5	1.8 ± 0.3
Mild intensity activities, min/day	241.8 ± 135.5	201.5 ± 107.4	332.3 ± 147.0	286.9 ± 124.4	143.7 ± 67.0
Moderate intensity activities, min/day	33.3 ± 44.7	30.0 ± 33.1	49.2 ± 50.8	38.8 ± 59.2	14.6 ± 14.8
Vigorous intensity activities, min/day	2.1 ± 5.1	4.2 ± 7.5	1.0 ± 2.7	2.7 ± 5.2	0.4 ± 1.5
Moderate + Vigorous intensity activities, min/day	35.2 ± 47.8	32.2 ± 39.2	50.2 ± 50.9	41.5 ± 62.4	15.0 ± 15.2
Time on body, min/day	98.9 ± 14.1	99.3 ± 0.9	99.6 ± 4.8	99.2 ± 6.7	79.1 ± 30.3

Data are presented as mean ± SD, unless otherwise stated.. *Abbreviations*: *Device 1* Smartwatch, *Device 2* Fitbit, *Device 3* Smartphone (Android), *Device 4* Smartphone (iOS)

The supervised series of 4 physical activity tasks confirmed an accuracy across the electronic devices usually used by patients (Table 3). For the static task of supine lying, the reference parameter was: 0 for the number of steps and 0 min for time duration of physical activity. When we compared the measured data with the reference parameter which was 0, we found no difference between the values for all electronic devices (Table 3). For the active tasks of stair-climbing, the reference parameter was 24 steps. We observed that the variability of measured steps was low and not significant (Table 3). Finally, for stationary cycling and walking, the reference parameter was 6 min of physical activity. All devices showed low variability for expected 6 min of physical activity (Table 3).

**Table 3:** Laboratory assessment of supervised series of 4 physical activity tasks using electronic devices

Variable	Reference	Device 1 (***n*** = 6)	Device 2 (***n*** = 6)	Device 3 (***n*** = 6)	Device 4 (***n*** = 6)
**Static task of supine lying**					
Duration of physical activity, min	0.0	0.0	0.0	0.17 ± 0.41	0.17 ± 0.41
Steps, number	0	7.83 ± 9.85	10.17 ± 9.11	6.33 ± 6.15	1.33 ± 3.27
**Active tasks of stair-climbing**					
Steps, number	24	19.83 ± 13.42	28.50 ± 8.85	23.33 ± 3.20	22.50 ± 1.87
**Active tasks of stationary cycling**					
Duration of physical activity, min	6.0	6.0 ± 0.0	3.75 ± 2.93	6.16 ± 3.18	5.88 ± 0.41
**Active tasks of walking**					
Duration of physical activity, min	6.0	6.0 ± 0.0	7.75 ± 3.5	6.33 ± 1.03	6.33 ± 0.52

Data are presented as mean ± SD, unless otherwise stated. *p <  0.05, differences between measured data with electronic devices and reference data. Reference data of supervised series of 4 physical activity tasks were established a priori*. Abbreviations*: *Device 1* Smartwatch, *Device 2* Fitbit, *Device 3* Smartphone (Android), *Device 4* Smartphone (iOS)

**Fig. 1:**
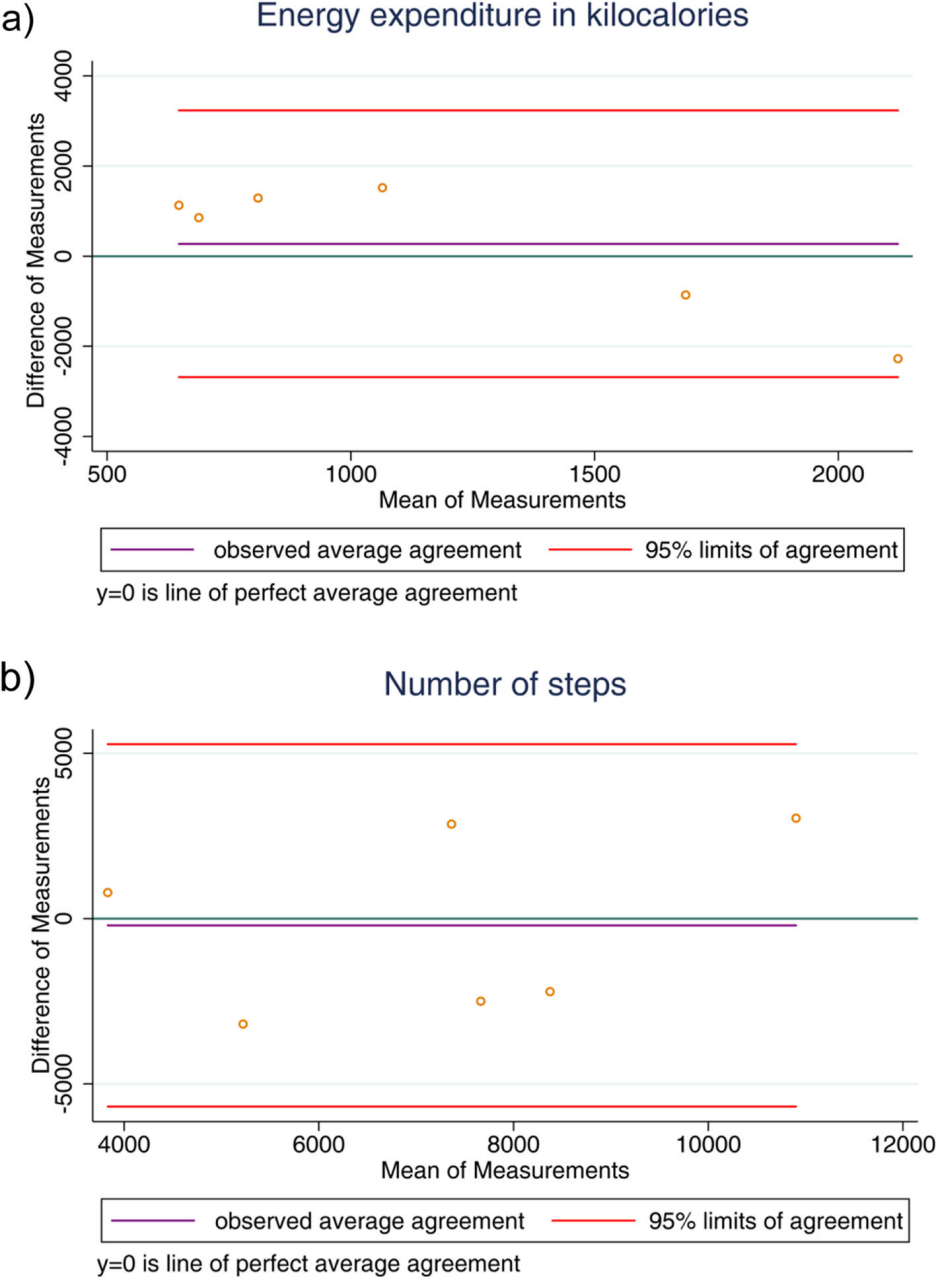
Fitbit Agreements. **a**: Average agreement and limits of agreement between SWA and Fitbit for active energy expenditure; **b**: Average agreement and limits of agreement between SWA and Fitbit for number of steps. (Authors’ source)

**Fig. 2:**
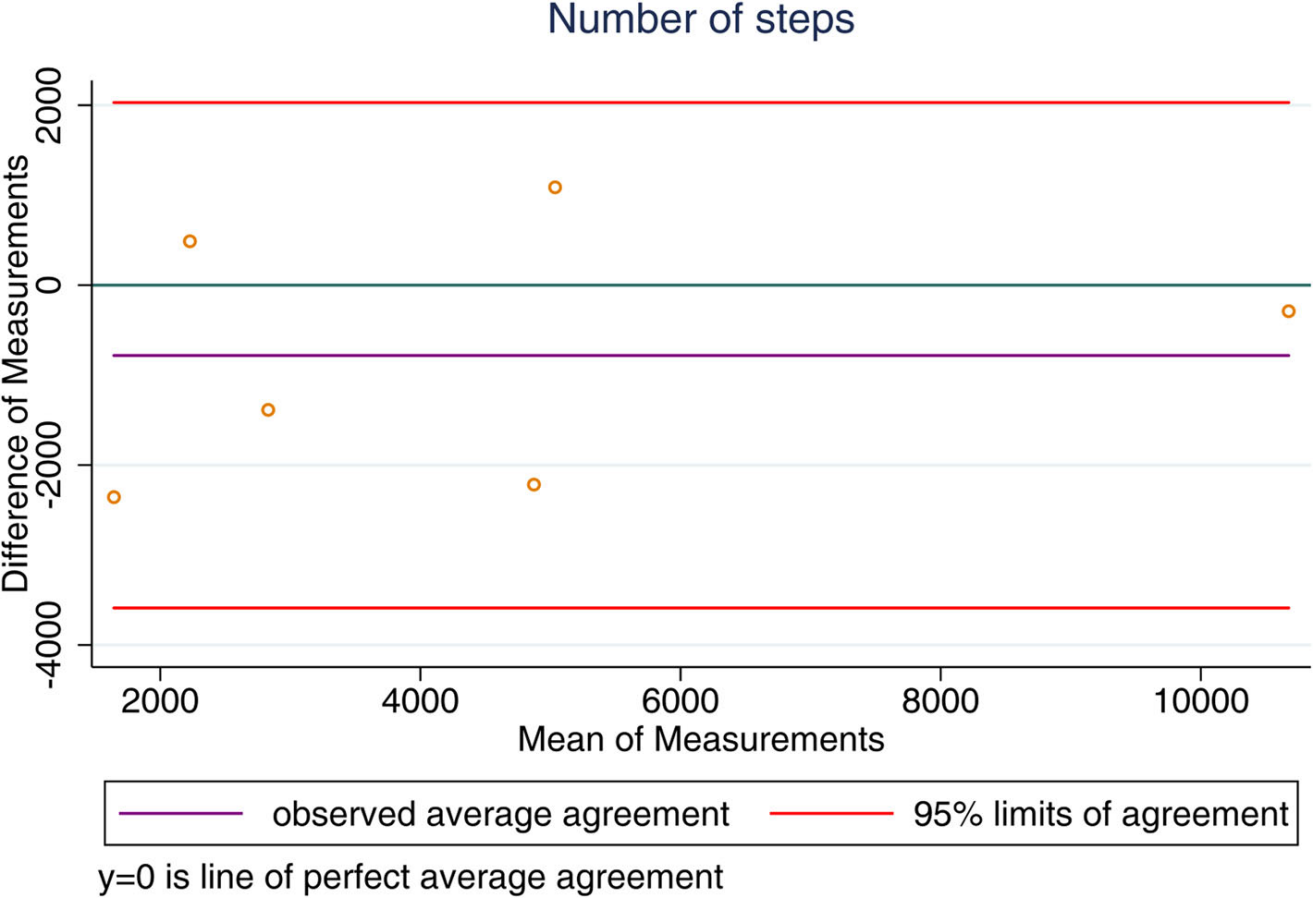
iOS Smartphone Agreements. Average agreement and limits of agreement between accelerometer SWA and iOS Smartphone for number of steps. (Authors’ source)

Results regarding differences between activity data reported by SWA and Smartwatch, Fitbit, Android Smartphone and iOS Smartphones are shown in Table 4. Among the studied devices, we found that there was no statistical difference between SWA and Fitbit for active energy expenditure (*p* = 0.143) and number of steps (*p* = 0.605). We observed that iOS smartphones were similar to SWA in monitoring the number of steps (*p* = 0.911). All other measurements showed significant differences between SWA and Smartwatch or Android smartphone (Table 4). The Bland-Altman method showed that the limits of agreements were quite large for all the devices. The Fitbit had the lower bias in the measurement of active energy expenditure and number of steps (Fig. 1 for Fitbit agreements and Fig. 2 for iOS Smartphone agreements; supplementary file for complete data agreements).

**Table 4:** Differences between accelerometer SWA and Smartwatch, Fitbit, Smartphone (Android) and Smartphone (iOS) on measuring daily physical activities

Variables			***P***-value
	**SenseWear Armband**	**SmartWatch**	
Active energy expenditure, Kcal	1197.8± 719.0	2025.8±1337.7	<0.001
Steps, number/day	9478.5±5251.1	14359.9± 8642.8	0.007
Duration Physical Activity, min/day	237± 129	311.7±199.3	< 0.007
	**SenseWear Armband**	**Fitbit**	
Active energy expenditure, Kcal	1302.2±458.4	1076.6±1281.5	0,143
Steps, number/day	7008.7±3945.1	7461.1±3482.0	0,605
Duration Physical Activity, min/day	382 ±157	70.2±48.7	<0.001
	**SenseWear Armband**	**Smartphone (Android)**	
Active energy expenditure, Kcal	1428.1±849.5	287.3±263.7	<0.001
Steps, number/day	9941.7±7056.2	8083.81±7683.5	<0.001
Duration Physical Activity, min/day	328±171	71.8±70.4	<0.001
	**SenseWear Armband**	**Smartphone (iOS)**	
Active energy expenditure, Kcal	692.7±323.0	173.8±161.1	<0.001
Steps, number/day	4512.9±3683.9	4937.2±4057.4	0.911
Duration Physical Activity, min/day	159±70	54.2±50.8	<0.001

*P<0.05, differences between activity data reported by SenseWear Armband andSmartwatch, Fitbit, Smartphone (Android) and Smartphone (iOS)

## Discussion

This pilot study is one of the first studies in CF to systematically assess the precision in daily life of commonly used electronic devices and compare them with the more conventional SWA. We showed good agreement between the SWA and Fitbit for parameters of physical activity such as active energy expenditure and number of steps. We also observed that iOS smartphones were similar to SWA in monitoring the number of steps, whilst Android smartphones and Smartwatch did not perform as well. If the data of this single pilot study are further confirmed by more extensive evidence, Fitbit devices and iOS smartphones could be introduced as a new strategy for assessing physical activity and lifestyle changes in the CF population, without increasing the burden of CF management.

In the last decade, there has been growing interest among the general public in monitoring daily PA using electronic devices. Activity trackers can improve awareness of activity levels and have been shown to motivate inactive patients with chronic diseases to become more physically active [18]. Recently, a range of activity trackers in various forms have been introduced to the market, including smartphone apps and wrist-worn devices with smartwatch-like functions. The user-friendliness of these commercial devices reported results in a high level of wear-time compliance [19, 20], while their monitoring of the various activities of daily life correlates well with that of clinical devices like accelerometers [20, 21]. Attempts to promote PA among patients suggest that the most effective strategies focus exclusively on PA [22], use behavioral approaches like feedback and goal setting [22, 23] and involve self-monitoring [24]. Patients prefer internet-based PA programs that are easy to use, with simple interactive features [24]. Recently, an internet-based program to assess and encourage participation of CF patients in PA was studied by Cox et al., who reported on its feasibility and acceptability [25]. Moreover, in CF studies, smartphones have been used to improve adherence [26].

In this pilot study, we evaluated for the first time the accuracy of smartphones and consumer activity wristbands in real life during activities of daily living in CF disease. We showed that the Fitbit and iOS smartphones provided daily PA measurements similar to the SWA accelerometer. Specifically, our data confirmed over 7 days, there was no significant difference between SWA and iOS smartphones for step count and no significant difference between SWA and Fitbit for number of steps and active energy consuming. Regarding the accuracy in measuring time spent doing physical activities, SWA and Fitbit did not correlate well. This could be related to the capacity of SWA to register activities of any grade of intensity while Fitbit only measures activities of moderate intensity or greater. This crucial component should be further investigated in a subsequent main study. If confirmed that certain electronic devices only record moderate physical activity and above, those devices may better reflect total time spent exercising, rather than total daily physical activity. When we investigated the precision of smartphones during activities of daily living, Android smartphones did not show good accuracy compared to the SWA. The lack of accuracy for Android smartphones group could be explained by the fact that CF patients reported to us that the smartphones were not being continually carried by them or the battery could have lost its charge. The similarity observed between iOS smartphones and SWA for monitoring the number of steps could be explained by the fact that the iOS group reported poor adherence on wearing SWA as showed by the lower value of the variable “time on body” (79% of SWA versus 99% of the other devices). As this is speculative, we believed that the potential precision of smartphones to assess PA should be investigated in further studies with larger number of patients. Among the activity wristbands, we found that Smartwatch did not correlate with SWA and all smartwatch data were higher compared with SWA. Patients in the Smartwatch arm reported that the device was comfortable and they were able to carry them continually. Three of the six patients reported going swimming and this was the reason they decided to use Smartwatch to routinely monitor PA. We can only speculate that the differences observed could be related to the fact that before swimming the SWA was removed as it is not waterproof.

The accuracy of Fitbit devices and iOS smartphones in assessing the number of steps is important for two reasons. First, steps represent PA that adults with CF perform regularly in their daily life (e.g., walking around the home or office, shopping, and walking from the parking lot). Second, taking 10,000 steps daily demonstrates the value of daily activity; individuals who achieve this are likely to fulfil World Health Organisation (WHO) recommendations of at least 150 min of above-moderate intensity PA each week [27]. Patients using the Fitbit commented that the device was comfortable and fashionable. They also reported good compliance due to its user-friendliness. Our results confirmed previous study, which showed that Fitbit measures step number during walking with acceptable level of accuracy [14]. Quantifying PA by an objective method is valuable for monitoring the positive effects of rehabilitation [2]. Although we recognize the small sample size is a limitation, the results of this pilot study are interesting as they are amongst the first to show evidence of the potential benefits of the use of Fitbit or iOS smartphones to measure PA in CF. If confirmed by larger studies, these electronic devices could become a future approach to monitor the activity levels in CF patients, using it both in clinical practice, in virtual clinic or in long-term follow up clinical trials. This is advantageous over current accelerometers, as these are burdensome, leading to decreased adherence and technical issues (e.g. connection issues for data sending, hardware incompatibility, etc.).

CF is characterized by episodes of pulmonary exacerbations. *Rosenfeld* et al. demonstrated that symptoms were more predictive of a pulmonary exacerbation than physical examination and laboratory values [28]. Physical activity during acute pulmonary exacerbation was investigated in several CF studies. The results showed that activity levels were lower in patients with acute pulmonary exacerbation compared with stable controls [29] and that more pulmonary exacerbations in the preceding year were associated with less active CF patients [30]. The availability of new electronic devices could evaluate daily physical activity and could be a new approach to monitoring a health decline that should represent an early sign of a CF exacerbation.

The present pilot study has important limitations. First, we involved a relatively small number of participants, particularly as the patients were divided into four groups. We acknowledge the possibility of type 2 error due to the small sample size, so only interesting observations can be made both for the laboratory supervised physical activity tasks and for home monitoring daily physical activities. Second, the heterogeneity of different types of electronic devices used routinely by the patients may have contributed to the heterogeneity of the group within the group. Then, our CF patients were adults thus it is possible that it did not fully represent habitual PA in a younger CF population. Finally, it is possible that our CF adults were less familiar with using electronic devices, rather than CF adolescents. Further research including similar manufacturer of devices and a larger cohort of children and adolescents will be needed to develop the role of electronic devices for exercise in CF throughout the full disease spectrum. Standardization of PA monitoring and reporting is essential for future research.

## Conclusion

In this pilot study of adults with CF and exercise monitoring, Fitbit and iOS smartphones performed well when compared with the accelerometer SWA. They are both able to accurately monitor step count and Fitbit also to accurately assess energy expenditure. As this is a pilot study that for definition is a smaller version of a proposed research study, these results could be used to refine the methodology and plan a definitive study. If confirmed by larger studies, Fitbit and iOS smartphones might have an important role in future exercise research studies in CF, especially because step count and energy expenditure are the recommended minimum standard for reporting PA. Finally, as the Fitbit is widely used in modern society, it has great potential to be easily integrated into an exercise program for CF patients who could find this highly motivating, although clearly further prospective studies of exercise interventions are required.

## Supplementary information

**Additional file 1.** **Supplemental File Figure 1.** Smartwatch Agreements. a: Average agreement and limits of agreement between SWA and Smartwatch for active energy expenditure; b: Average agreement and limits of agreement between SWA and Smartwatch for duration of physical activity (data available for four patients); c: Average agreement and limits of agreement between SWA and Smartwatch for number of steps. **Supplemental File Figure 2.** Android smartphone Agreements. a: Average agreement and limits of agreement between SWA and Android smartphone for active energy expenditure; b: Average agreement and limits of agreement between SWA and Android smartphone for duration of physical activity; c: Average agreement and limits of agreement between SWA and Android smartphone for number of steps. **Supplemental File Figure 3.** iOS smartphone Agreements. a: Average agreement and limits of agreement between SWA and iOS smartphone for active energy expenditure; b: Average agreement and limits of agreement between SWA and iOS smartphone for duration of physical activity. **Supplemental File Figure 4.** Fitbit Agreements: Average agreement and limits of agreement between SWA and Fitbit for duration of physical activity (data available for three patients).

## Data Availability

The datasets used and/or analyzed during the current study are available from. the corresponding author on reasonable request.

## Abbreviations

CF: Cystic Fibrosis
BMI: Body mass index
FEV1: Forced expiratory volume in 1 s
PA: Physical activity
SWA: SenseWear Pro3 Armband
6MWT: 6-min walk test
METS: Metabolic equivalents
