# Supplementary material for: New strategies of physical activity assessment in cystic fibrosis: a pilot study
Source: BMC Pulm Med. 2020 Oct 30;20:285. doi: 10.1186/s12890-020-01313-5 (PMC7599110; doi:10.1186/s12890-020-01313-5)

**Assessment of daily physical activity*.***

***Characteristics of the multi-sensor armband (SenseWear Pro3 Armband)***

The SenseWear Pro3 Armband was positioned on the dominant upper arm over the triceps muscle at the midpoint between the acromion and olecranon processes. Patients wore the armband for seven full consecutive typical days (including 5 weekdays and 2 weekend days) when they were at home. Patients were instructed to wear the armband day and night and only to remove it for bathing or showering. Data are reported as the average of 7 days. It was reported in CF that 5 days monitoring was enough to assess habitual PA and PA levels were similar through the week (i.e., weekdays versus weekend days) [1,2]. The sensor contained a biaxial accelerometer, galvanic skin response sensor, heat flux sensor, skin temperature sensor, and a near-body ambient temperature sensor from which the data were stored every minute. Using specific software (version 6.1), these variables, as well as body weight, height, handedness and smoking status (smoker or non-smoker), were used to estimate energy expenditure (EE). Several aspects of EE, including total energy expenditure (TEE) and active energy expenditure (AEE) were also calculated. The outputs obtained from the armband were total physical activity duration, number of steps, time lying down, sleep duration and intensity of PA, expressed in metabolic equivalents (METS). The time (min) spent in PA at different intensities (mild, moderate, vigorous) and the definitions for activity levels based on METS were those used by *Troosters et al.* [1]. The time (in min) spent with an energy expenditure of >3 METS was considered “mild” activity (e.g., walking at normal walking speed, carrying out light household work), time spent at >4.8 METS was considered “moderate” activity (e.g., brisk walking or cycling) and activities with an energy expenditure of >7.2 METS were considered “vigorous” (e.g., running or activity with training effects when applied for a sufficient length of time and at an appropriate training frequency).

REFERENCES

1. Troosters T, Langer D, Vrijsen B, Segers J, Wouters K, Janssens W, et al. Skeletal muscle weakness, exercise tolerance and physical activity in adults with cystic fibrosis. Eur Respir J 2009;33:99–106.
2. Savi D, Quattrucci S, Internullo M, De Biase RV, Calverley PM, Palange P. Measuring habitual physical activity in adults with cystic fibrosis. Respiratory Medicine 2013;107(12):1888–94.

**Supplemental File Figure 1.** Average agreement and limits of agreement between accelerometer SWA and Smartwatch for: a) active energy expenditure; b) duration of physical activity (data available for four patients); c) number of steps.


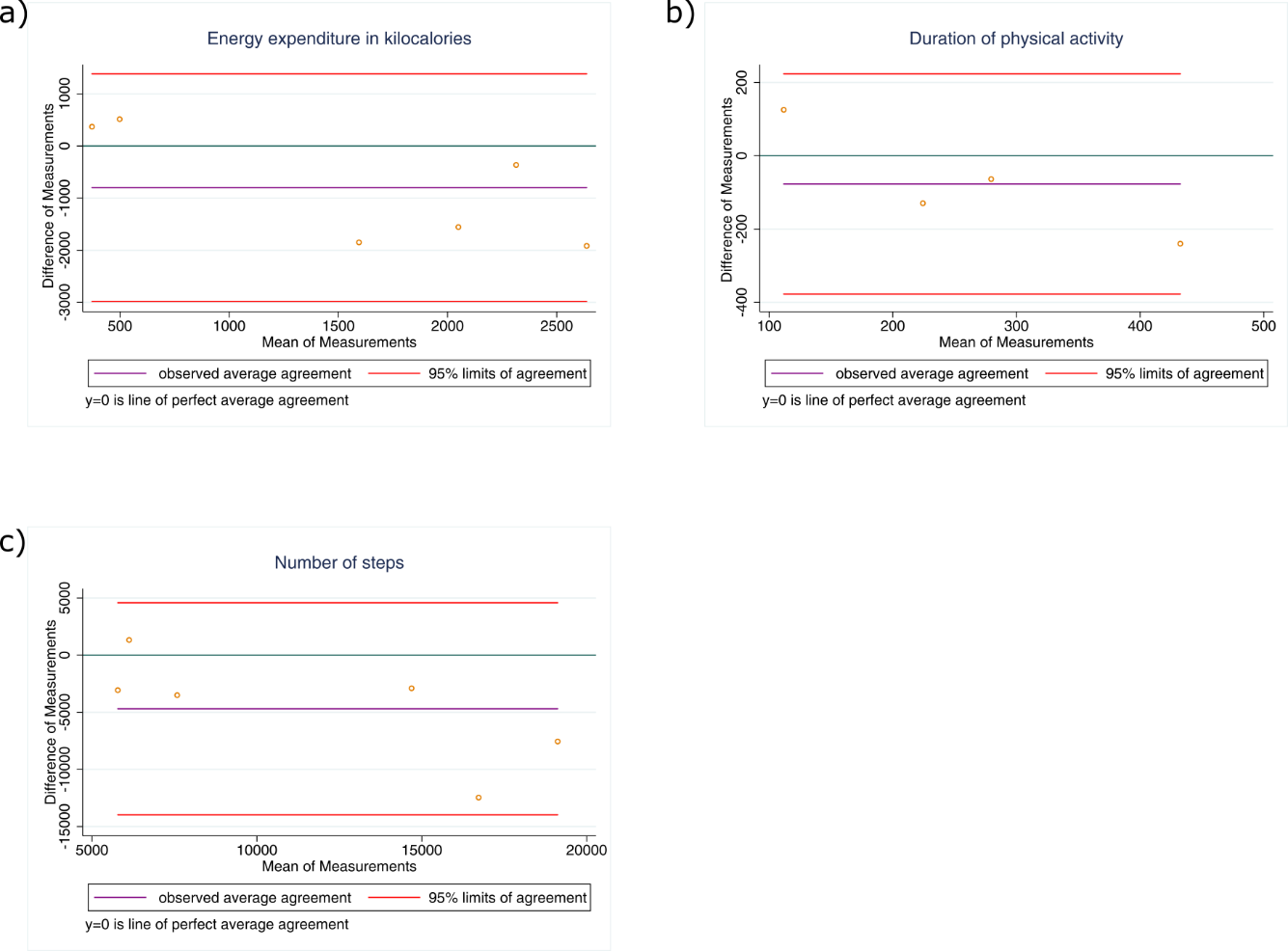


**Supplemental File Figure 2.** Average agreement and limits of agreement between accelerometer SWA and Android smartphone for: a) active energy expenditure; b) duration of physical activity; c) number of steps.

a)


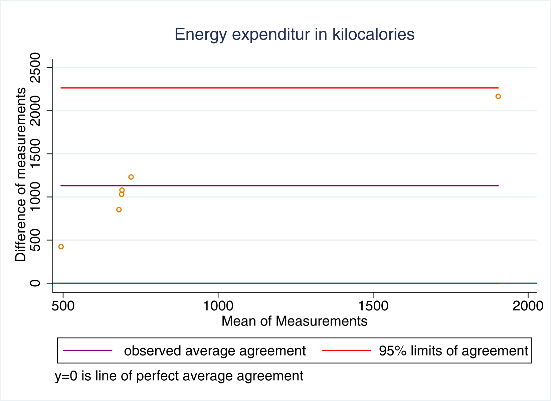


b)


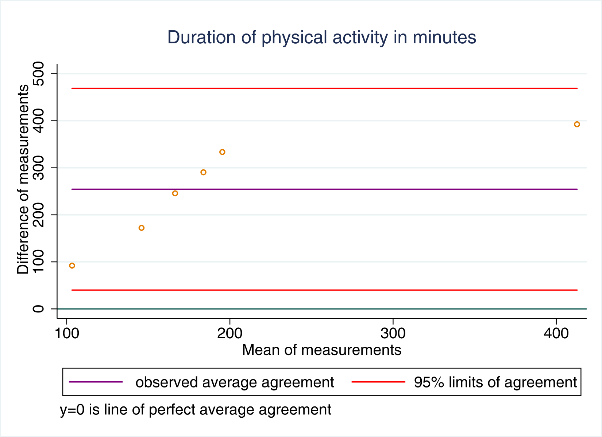


c)


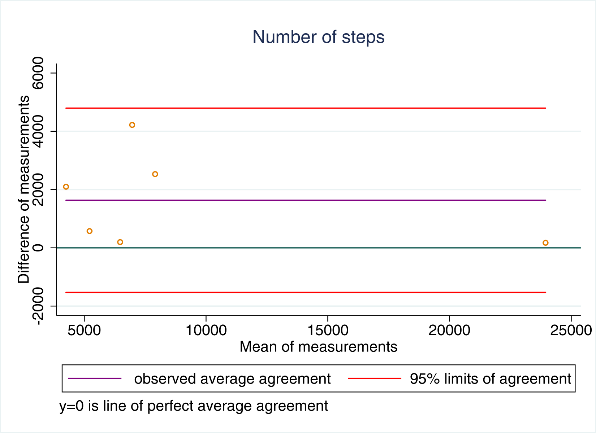


**Supplemental File Figure 3.** Average agreement and limits of agreement between accelerometer SWA and iOS smartphones for: a) active energy expenditure; b) duration of physical activity.


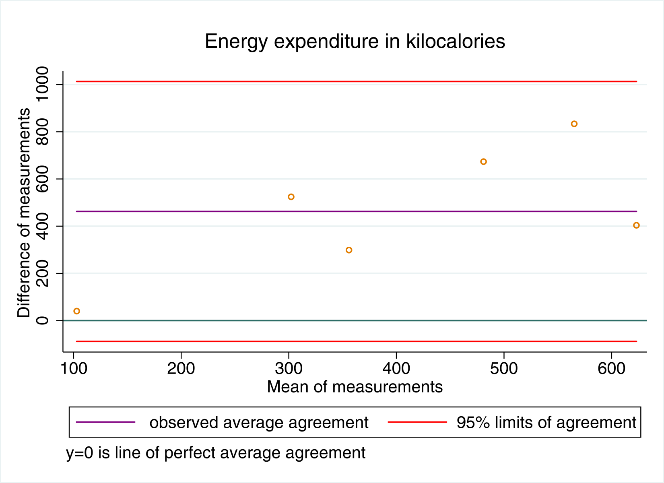


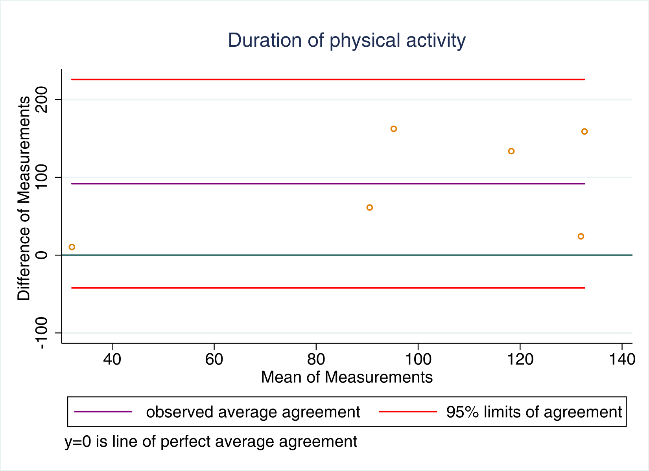


**Supplemental File Figure 4.** Average agreement and limits of agreement between SWA and Fitbit for duration of physical activity (data available for three patients).


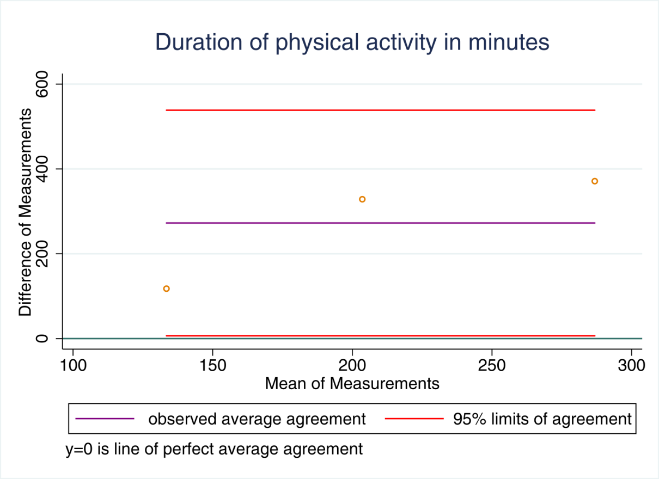

Supplement: Supplementary file 1 — Additional file 1. Supplemental File Figure 1. Smartwatch Agreements. a: Average agreement and limits of agreement between SWA and Smartwatch for active energy expenditure; b: Average agreement and limits of agreement between SWA and Smartwatch for duration of physical activity (data available for four patients); c: Average agreement and limits of agreement between SWA and Smartwatch for number of steps. Supplemental File Figure 2. Android smartphone Agreements. a: Average agreement and limits of agreement between SWA and Android smartphone for active energy expenditure; b: Average agreement and limits of agreement between SWA and Android smartphone for duration of physical activity; c: Average agreement and limits of agreement between SWA and Android smartphone for number of steps. Supplemental File Figure 3. iOS smartphone Agreements. a: Average agreement and limits of agreement between SWA and iOS smartphone for active energy expenditure; b: Average agreement and limits of agreement between SWA and iOS smartphone for duration of physical activity. Supplemental File Figure 4. Fitbit Agreements: Average agreement and limits of agreement between SWA and Fitbit for duration of physical activity (data available for three patients). [file 12890_2020_1313_MOESM1_ESM.docx]
